# Leveraging integrated data for program evaluation: Recommendations from the field

**DOI:** 10.1016/j.evalprogplan.2022.102093

**Published:** 2022-12

**Authors:** Sharon Zanti, Emily Berkowitz, Matthew Katz, Amy Hawn Nelson, T.C. Burnett, Dennis Culhane, Yixi Zhou

**Affiliations:** University of Pennsylvania, Actionable Intelligence for Social Policy, United States

**Keywords:** IDS, Integrated Data System, AISP, Actionable Intelligence for Social Policy, OPRE, Office of Planning, Research & Evaluation, T&TA, Training and Technical Assistance, ERP, Encampment Resolution Pilot, NFP, Nurse Family Partnership, Cross-sector data linkage, Integrated data systems, Administrative data reuse

## Abstract

Use of administrative data to inform decision making is now commonplace throughout the public sector, including program and policy evaluation. While reuse of these data can reduce costs, improve methodologies, and shorten timelines, challenges remain. This article informs evaluators about the growing field of Integrated Data Systems (IDS), and how to leverage cross-sector administrative data in evaluation work. This article is informed by three sources: a survey of current data integration efforts in the United States (U.S.) (N=63), informational interviews with experts, and internal knowledge cultivated through Actionable Intelligence for Social Policy’s (AISP) 12+ years of work in the field. A brief discussion of the U.S. data integration context and history is provided, followed by discussion of tangible recommendations for evaluators, examples of evaluations relying on integrated data, and a list of U.S. IDS sites with publicly available processes for external data requests. Despite the challenges associated with reusing administrative data for program evaluation, IDS offer evaluators a new set of tools for leveraging data across institutional silos.

## Highlights

• Using linked administrative data benefits social program and policy evaluation.
• Over 93 data sharing and integration efforts currently exist across the U.S.
• 15 of these sites have data request processes for external evaluators.
• There are nine key recommendations for how evaluators can work with these sites.

## 1 Introduction

The reuse of administrative data is now commonplace in both the private and public sectors, and tools and approaches are rapidly expanding. Researchers, service providers, and policymakers are recognizing how data collected as part of routine public and social service operations can be reused to better understand the impact of programs and policies. A 2017 report from the United States (U.S.) Office of Planning, Research & Evaluation (OPRE) considered key issues of data access in response to increasing interest among policymakers to address “pressing, policy-relevant questions” (Maxwell, 2017, p. 1). Likewise, a partnership between Chapin Hall at the University of Chicago and the U.S. Census Bureau explored the utility of existing government data for analysis and evaluation, and piloted six test projects linking local, state, and federal datasets; input for the project was received from all levels of government, universities, research firms, and other nonprofits (Goerge et al., 2017). The field of social work, among others, has specifically identified the importance of reusing linked administrative records as part of its commitment to “harnessing big data for social good” (Coulton et al., 2015, p.3). As interest in utilizing linked administrative data for evaluation grows, so do the opportunities and methods available to ethically access, link, and analyze these data to better understand what works for individuals, families, and communities.

The evaluation field has long used administrative data to conduct impact and process evaluations for programs and policies (Barnow, 1986, Berger et al., 2013, Lundgren et al., 2003), demonstrate longitudinal program outcomes (Murphy et al., 2017, Oh et al., 2020), supplement and triangulate self-report data (Courtney et al., 2007, Evans et al., 2011, Lemieux et al., 2017), and even predict which program participants will experience a desired or undesired outcome (Kim et al., 2020). Such studies highlight the advantages of using administrative data in evaluation and program planning work, including the availability of historical data, reduced time and resources for data collection, and large sample sizes and comparison groups. However, the challenges of working with administrative data are also well documented (Green et al., 2015, Harron et al., 2017, Johnson et al., 2015, Netten et al., 2017). Decisions around privacy preservation techniques, database type and structure, and how to marry legacy systems with new ones further complicate the process of moving towards data integration (Actionable Intelligence for Social Policy, 2021, Bargh et al., 2016, Card et al., 2010, Goerge, 2018, Van den Braak et al., 2016). Moreover, not only is gaining access to relevant data often a long and arduous process due to privacy and security protocols, but once data are accessed, data quality and operationalizing concepts with the available data present additional challenges (Harron et al., 2017).

In recent years, as government, health care, education, and other public domains have turned to data integration, there has also been increased focus on surfacing solutions to the challenges of administrative data reuse.^1^ At a high level, integrated data link individual-level data across agencies and/or programs in order to provide a more holistic, longitudinal view of clients, families, and communities than is available using any single agency data source, and can be used to inform program planning and policymaking (Hawn Nelson et al., 2020b). An Integrated Data System (IDS) is a formalized effort that enables the routine sharing and reuse of cross-sector administrative data with strong governance and legal agreements in place^2^, and is also sometimes referred to as a data hub, State Longitudinal Data System, data collaborative, or data trust (Hawn Nelson et al., 2020b, Goerge, 2018). Based on a comprehensive scan of efforts across the U.S. there are over 90 state and local jurisdictions, non-profits, and universities in the U.S. currently operating some type of data integration effort (Berkowitz et al., 2020).

Actionable Intelligence for Social Policy (AISP) currently coordinates a network of IDS sites that are continually refining their processes, engaging in peer learning to develop best practices, and actively reusing administrative data for evaluation, research, and planning purposes (Culhane et al., 2010). AISP, housed at the University of Pennsylvania’s School of Social Policy & Practice, supports government agencies and their partners in sharing and integrating cross-sector data to improve insights and evaluate social policy and practice. As evaluations seek to better understand the lived experiences of individuals, families, households, and neighborhoods, IDS offers evaluators a new set of tools for leveraging data across institutional silos.

The following sections of this paper explore the context and history of data integration in the U.S. and the current state of this emerging field. Then, drawing on a recent national landscape survey, expert interviews, and AISP’s 12+ years of work in the data integration field, concrete insights and recommendations are provided for evaluators interested in working with IDS data, followed by examples of “Work in Action'' that demonstrate how this work can be carried out successfully and collaboratively. Finally, a detailed list of IDS sites that work with external evaluation partners is included in order to foster awareness of available individual-level, linkable data and new potential partnerships.

## 2 A note on the United States context

The recommendations shared in this paper were developed within the U.S. context, where legal and governance challenges often present the largest barrier to evaluators accessing administrative data from human service programs. This arises in large part due to the country’s highly decentralized government that is resource constrained. Funding for human service programs in the U.S. is typically distributed and governed at the state and local level (Urban Institute, 2021). The federal government distributes grants to these jurisdictions, who usually bring matching funds and further supplement resources through taxation, public-private partnerships, and licensing/fees. As a result of this decentralized structure, human service data often reside at the state or local level. However, accessing these data as an evaluator can be complex given the misalignment across jurisdictions on data infrastructure, legal access procedures, and governance (Abazajian and Kleykamp, 2020, United States Government Accountability Office, 2020). In addition, the U.S. is currently in a period of austerity, which impacts health and human service programs through spending cuts that hamper both government services themselves and agencies’ ability to fund robust evaluations (Reich et al., 2017, Results for America, 2021).

The field of data integration is growing in the U.S., most notably at the state and local level, with more and more agencies identifying best practices for aligning data access procedures and scaling data infrastructure within a constrained fiscal environment. While IDS and the capacity to share and link data ethically can support cost-savings and increased efficiencies for state and local jurisdictions (Hawn Nelson et al., 2020b), scaling an IDS requires substantial time and resource investments upfront (Actionable Intelligence for Social Policy, 2021). There is emerging guidance, standards, and training from AISP and other data integration organizations to address this dilemma. This paper draws on work from U.S. states and localities currently leveraging data for policy and program evaluation to offer key lessons learned for evaluators to succeed within a decentralized system of government and during times of fiscal constraint.

## 3 A brief history of administrative data integration efforts in the U.S

Data integration in U.S. academic literature dates back to Dunn (1946), who articulated the importance of linking vital statistics records in order to inform public health and social welfare programming. While data integration is clearly not new and not reliant upon digital formats, the complexities and capabilities associated with it continue to grow as data become “big,” records become digitized and machine readable, and disparate data can be linked through data integration techniques and technologies.

In the 1970s, a statistician for the State of South Carolina, Walter “Pete” Bailey, pioneered state-level data integration for research and policy using health records (Kitzmiller, 2014). Integrating data across government agencies allowed South Carolina to better understand disease prevalence, develop population definitions, conduct needs assessments, measure program outcomes, and ultimately leverage their vast stores of administrative data for social good (Bailey, 2003). For instance, the state leveraged their IDS capacity to evaluate the impact of a pilot psychiatric telehealth initiative that aimed to address the shortage of rural mental health professionals. Findings showed improved patient outcomes compared to treatment as usual, which led South Carolina to steadily scale the telehealth program to additional rural hospitals to reach more individuals in need of psychiatric services (Cooner, 2018).

Additional IDS efforts at the state and local level started popping up in the 1990s, such as Chapin Hall at the University of Chicago and Allegheny County (Pittsburgh, Pennsylvania) Human Services. In Philadelphia, University of Pennsylvania professors Dennis Culhane and John Fantuzzo established an IDS that linked data on the city’s children that could be used for research and evaluation purposes with strong security and privacy measures in place. The University housed the IDS with oversight from city leaders until 2008, at which point the data infrastructure transitioned to the office of the Philadelphia Deputy Mayor for Health and Opportunity. Around the same time, Culhane and Fantuzzo received funding from the MacArthur Foundation to establish AISP and begin exploring the broader, national IDS landscape. Through this process, Culhane and Fantuzzo identified eight existing IDS sites across the U.S., and more importantly, discovered that despite some organizational and operational differences, these disparate jurisdictions experienced similar core challenges in developing their data integration efforts^3^. Since 2008, AISP has cultivated a national network of IDS sites, created and disseminated training, and developed best practices across a variety of data integration topics in order to strengthen the use of integrated data in evaluation, planning, policymaking, and research (Actionable Intelligence for Social Policy, n.d.a).

As a result of these and other efforts, the field of data sharing and integration continues to grow and mature. In 2017, AISP launched its Learning Community initiative, a formal training and technical assistance (T&TA) program developed in response to emerging needs at the state and local level for support in building capacity for routine data linkage (ROI Impact Consulting, 2019). Since its launch, this initiative has trained 18 jurisdictions and grown the formal AISP Network to 35 active members. In 2020, AISP identified 93 sites integrating civic data across the U.S. and conducted a national landscape survey to gain insights about the current state of the field. Among respondents (N=63), nearly half (46%) launched their IDS effort in the last five years (Berkowitz et al., 2020). Additionally, roughly three in four of these same efforts have governance processes in place—a notable improvement in standardization of best practices among sites over the last three years, according to experts in the field (Berkowitz et al., 2020). Such formalized procedures and systems help facilitate the use of IDS to build and use evidence for program planning and evaluation.

Administrative data reuse to inform policy and practice has become a stated priority at every level of government in the U.S. (Commission on Evidence-Based Policymaking, 2017, National Association of Counties, 2018, Smart Cities Council of North America, 2017, State Data Sharing Initiative, 2018, United States, Executive Office of the President, 2019). Yet the field of data integration in the U.S. is newly emerging and, as such, federal guidance is nascent, and questions of data governance and use have remained firmly in the hands of state and local governments. In the past ten years, Chief Data Officer positions have been established in over half of U.S. states, though roles and responsibilities vary from state to state (Kleykamp, 2020). This trend also extends to cross-discipline academic studies, as is evidenced by the increasing number that use integrated administrative data to better understand pressing social issues and long-term outcomes of individuals and population subgroups (Chetty et al., 2014). Integrated data are being leveraged for planning and evaluation purposes, including routine program evaluation and descriptive reports (Crampton et al., 2020, Danielson et al., 2020, Metraux et al., 2019) as well as longitudinal, cross-sector studies (Culhane et al., 2019, Putnam-Hornstein et al., 2013).

Linked administrative data held within IDS can elevate both small and large-scale evaluation studies by reducing the time, funding, and energy that would otherwise be required to conduct original data collection and linkage. Cost-savings for research and evaluation are frequently cited by data sharing advocates as well as state and local stakeholders as a key benefit of data integration capacity (Coalition for Evidence-Based Policy, 2012, Culhane, 2016, Data Quality Campaign, 2019, Hart and Potok, 2020, Kitzmiller, 2013, Lee et al., 2015, Results for America, 2020). Moreover, the use of linked data enables states and localities to conduct more granular research. For example, a single program evaluation with access to vast stores of linkable administrative data can compare program participant outcomes to the broader population across social programs and yield more actionable insights. The use of IDS can also mediate common issues in survey research, like insufficient sample sizes and comparison groups, and low survey response rates, as administrative data collection routinely captures population-level data.^4^ In addition, rapid evaluations using embedded RCT or quasi-experimental designs become more feasible using existing infrastructure, rather than establishing new data flows. Evaluators are able to access existing data, rather than collecting their own, and construct demographically similar comparison groups for analysis, depending on system and staff capacity.

While IDS give evaluators the potential to combine data across sectors to deepen program insights, it is important to remember that administrative data are collected primarily for operational and not evaluation purposes. As a result, these data come with limitations related to the availability of information within administrative datasets, data quality issues, and barriers to access. Working with these data requires in-depth knowledge of both the IDS and the specific administrative datasets one seeks to access in order to ethically and appropriately reuse them for evaluation purposes.

## 4 Methods

This paper is informed by three sources of knowledge: a national survey of current data integration efforts; informational interviews with data sharing and integration experts; and, AISP’s internal knowledge cultivated through 12+ years of work in the U.S. and international IDS field.

### 4.1 National survey

In January of 2020, AISP conducted a national survey of data integration efforts across the US. The online survey was distributed to 93 data sharing efforts that varied in model, purpose, and size to capture a comprehensive understanding of the data sharing landscape and to identify their current policy priorities and data holdings. The survey, with a 67% response rate, included numerous qualitative questions and focused on the core components of IDS—governance processes, legal agreements, technical approaches, staffing and resource capacity, and impact on policy and programs (Berkowitz et al., 2020). AISP is confident that the sample selection process was comprehensive and largely successful in capturing the landscape of data sharing and integration efforts nationally. Of the 63 respondents, 62% are members of the AISP Network or T&TA Learning Community, while the remaining 38% are not formally affiliated and were identified using snowball sampling with key informant input.

Survey respondents were not compensated for their participation. Findings from the landscape analysis survey were developed into an internal report for the philanthropic funder. Both the national survey and unpublished report inform this paper—in particular, the “A Brief History of Administrative Data Integration Efforts in the U.S.” and “How to Access Administrative Data for Integration” sections.

### 4.2 Informational interviews

The authors of this paper conducted eight 30-minute informational interviews with experts who use IDS in applied settings. Informants represented a diverse group of professionals and practitioners from public and private universities, state and local jurisdictions, and nonprofit agencies. They were chosen based on their experience as IDS administrators working with program evaluators. Their primary job duties also varied, including community engagement, technical assistance, and traditional research and analysis roles. There was notable geographic diversity among those interviewed, consistent with the geographic diversity of IDS in the U.S. (Actionable Intelligence for Social Policy, n.d.b). At least two authors participated in each interview and recorded detailed notes. Notes from each interview were then reviewed by multiple authors and summarized into key themes, which the authors then discussed and triangulated with survey findings. Interviewees were not compensated for their participation.

### 4.3 Internal expertise

Collectively, AISP staff have over four decades of experience working as a core part of an IDS and/or in support of IDS development across the U.S. Notably, this experience includes leading 30+ long-term site-based technical assistance contracts focused on facilitating cross-sector data access and use. This article draws upon this extensive experience, and the resources AISP has developed in the past 12 years, including research publications, white papers, toolkits, evaluations, briefs, and case studies. All of these sources of knowledge informed the interpretation and presentation of informational interviews conducted with field experts as well as the conclusions of this article.

## 5 How to work with integrated data systems

Though IDS provide the potential for evaluators to gain access to more holistic data and navigate the core challenges of reusing administrative data, there are important considerations to keep in mind when pursuing this work. The following recommendations are intended to prepare evaluators for approaching the utilization of IDS and building successful partnerships with those that operate them. Drawn from the U.S. context, these insights are most instructive to researchers and evaluators working with state or local jurisdictions operating under a decentralized government structure and during times of decreased government services funding. For these reasons, it is important to note that no two IDS sites are alike, and unique processes and challenges should be expected to arise.

### 5.1 Understand how the IDS is managed

**Fig. 1:**
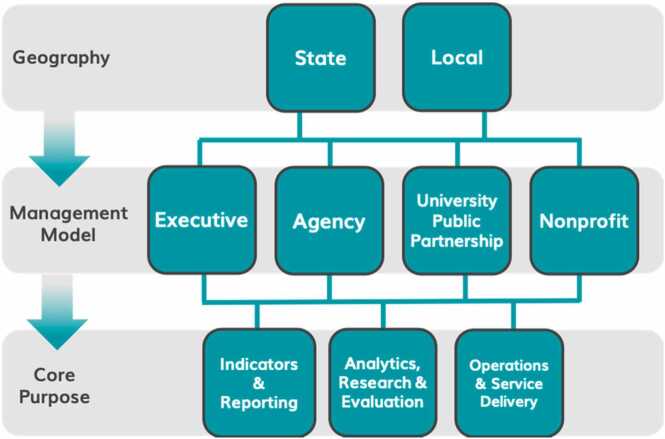
Taxonomy of integrated data systems.

While each IDS is unique, there are some distinctions across among models when considering three key organizing principles: geography (either at the state or local level), management model (or, the kind of organization that hosts the system and leads governance), and the goal or purpose (or, how and for what an IDS is used) (Berkowitz et al., 2020). AISP developed a taxonomy and quality framework around these dimensions to help capture and better understand similarities and differences among IDS efforts across the country, shown in Fig. 1 (Actionable Intelligence for Social Policy, 2021).

For evaluators, understanding the management model and host organization of an effort is particularly helpful to anticipate common practices and challenges for a site; each is explored below in more detail.

#### 5.1.1 Executive-led IDS sites

Executive-led IDS are hosted by an executive-level office (e.g., Mayoral office, state Office of Budget and Management) and are, therefore, often closely connected to the leadership’s policy agenda. This proximity to decision makers is often a key advantage for external partners, such as evaluators, who intend to meaningfully inform policy and programs through their work. However, working with executive-led sites also necessitates catering evaluation proposals to align with the current policy priorities, which can shift with elections and administration changes.

Additionally, these are often well-resourced and well-established IDS sites, which can translate to benefits for evaluators, including standardized procedures for data access and use, dedicated staff to manage data request processes, and the long-term sustainability of the IDS. Further, like most sites hosted by government entities or universities, executive-led efforts have insurance coverage and security protocols to manage data incidents or breaches.

#### 5.1.2 Agency-led IDS sites

Agency-led IDS sites are hosted by a large umbrella agency (e.g., Department of Health and Human Services, Department of Education). These can also be housed in centralized IT offices or standalone agencies designed for the sole purpose of governing, integrating, and analyzing administrative data. Proximity to both people represented within the data and practitioners implementing services may enhance social license for data use and present more opportunity for authentic engagement with the community in evaluations^5^. Agency-led sites also tend to have more experience working with evaluators as part of their routine operations, but their sharing and access processes may not be as straight-forward as in executive-led sites. In addition, reticence to work with external evaluators may arise in agencies that have experienced public backlash around data use, as agencies often receive the brunt of such criticism.

#### 5.1.3 University-public partnership IDS sites

University-Public Partnership sites are hosted by a university, often in collaboration with or in service of government agencies at the state or local level. External evaluators are more likely to go through formal review processes, such as IRBs, ethical reviews, and/or community reviews, when working with these sites. These processes take time, making University-Public Partnership sites inherently less flexible to urgent data needs. Flexible staffing, including graduate interns and workers, is a benefit of working with university-hosted efforts. Technical and content xpertise of site personnel may also help evaluators flesh out research questions, identify appropriate data sources, and provide additional context to improve quality of findings and to develop actionable insights. Evaluators associated with the university system hosting the IDS may find it easier to get access to data due to greater knowledge of the IDS and familiarity with formal review processes.

#### 5.1.4 Nonprofit-led IDS sites

Nonprofit-led sites are hosted by a non-profit agency or backbone organization (e.g., United Way), often with external partners (private, university, agency, or other) performing other key functions. This is an uncommon but emerging model, particularly among local IDS efforts. Evaluators approaching these sites are less likely to encounter established processes, but in turn, may find fewer barriers to data access and deeper connections to the communities impacted by evaluation work. Another unique consideration is the data security and liability protocols in place. Universities, government agencies, and other large institutions are accustomed to handling large, sensitive data sets and have insurance to cover data breaches and security incidents. Nonprofits without institutional partnerships may be less likely to offer insurance provisions, meaning that the evaluator must understand the nonprofit’s position and establish security protocols as needed.

### 5.2 Get involved in early community outreach efforts that are developing or informing integrated data work

This can include attending committee meetings, formally joining a task force, or offering to lead or facilitate community forums on the topic of data integration (Future of Privacy Forum & Actionable Intelligence for Social Policy, 2018). Early outreach and participation can provide a pathway for evaluators to become known and trusted entities by IDS data partners and demonstrate genuine interest in working collaboratively with the community. Moreover, this strategy sets up evaluators to gain an in-depth understanding of the IDS’s governance process, legal requirements, and available data so that they are poised to successfully propose future projects and respond to grants aligned with site interests. Evaluators can take this approach with IDS sites that are not yet operational or open to external evaluation, and in doing so, bring valuable external input to IDS development. This strategy also sets up the evaluator to be a natural, long-term partner and build relationships across the community.

### 5.3 Gaining access to IDS data is not quick and easy; set realistic expectations

The process for gaining access to IDS data is often long and bureaucratic, stemming from the necessary privacy and security protections for individual-level, cross-agency data. It is important for evaluators and their funders to set the expectation that gaining data access may take longer than desired. There may also be strict limitations on which data can be requested from the IDS and not all data requests will be approved. Therefore, flexibility is key, as is understanding that timelines and processes are not fixed.

### 5.4 Understand the historical, cultural, and political context in which the IDS exists before approaching with a project idea

Beyond exploring an IDS’s website for mission, vision, values, process overviews, data request forms, and other public-facing material, evaluators should build relationships with the people behind the IDS in order to glean relevant insight—most of which is simply not available online. For example, sites may reject data requests not aligned with their stated priorities; political tensions may exist between programs sharing data with the IDS; or past harms resulting from improper data uses may still inhibit trust in sharing data with outside evaluators. This may also be true of communities and people represented within datasets who have been harmed by evaluations that did not include their input, perspective, or needs (Chicago Beyond, 2018, Hawn Nelson et al., 2020a). These are all important insights that can help evaluators more thoughtfully propose IDS projects and often require developing interpersonal relationships with key IDS stakeholders.

Further, because IDS use administrative data, there is often bias baked into the datasets. Legacies of racism in policy and programs have led to overrepresentation of Black, Indigenous, brown, and other communities of color in administrative datasets. Without careful attention to racial equity, administrative data reuse comes with risk of reinforcing racism and inequitable resource allocation, access, and outcomes. Evaluators should strive to center racial equity and consider potential harm to vulnerable communities in their proposals. More IDS sites are considering risks and consequences of using biased and racist data as well and may increasingly call for explicit consideration of race and related vulnerabilities in external evaluation proposals.

To start, figure out if the IDS has a point person who manages evaluation requests and contact them to field initial questions and gain a more in-depth background on the IDS and its priorities. As noted previously, joining committees or attending public forums related to the IDS can also be a constructive way to understand the context in which the IDS operates.

### 5.5 Get to know the IDS’s governance and legal processes for working with external partners; then, follow them

Governance can be thought of as the “rules of the road” for working with the IDS—it is the policies and procedures that determine how data are managed, used, and protected. Legal processes go hand-in-hand with governance to create a clear structure that guides ethical cross-agency data sharing, including steps like signing data sharing agreements and creating provisions for protecting personally identifiable information (Gibbs et al., 2017). This process reassures agencies sharing data to the IDS that their data will be protected and used ethically. The core IDS data partners spend considerable time and energy setting up governance and legal structures to reduce future barriers to using data while also mitigating the risk of data privacy violations. They become experts in these functions, and evaluators can leverage that expertise by following established structures to propose projects, request data, and setup data sharing. This not only removes work from the evaluator’s plate but can also serve to cultivate a positive relationship with the IDS for long-term partnership.

### 5.6 Thoroughly understand the data

This recommendation applies to both the data being requested from the IDS as well as data the evaluator brings to the project. For data requested, evaluators should begin by doing as much independent research as possible on which data are available within the IDS and how those data align with their potential project proposal. Then, it is critical to review all documentation and discuss each data field in detail with those who already work closely with the data (which may be a combination of IDS administrators and/or agency/program staff), paying particular attention to issues around data collection, cleaning, and quality. Administrative data are not collected for evaluation purposes and, therefore, not all data fields will be appropriate for reuse. Working with administrative data may also necessitate creativity and flexibility in how variables are operationalized since evaluators cannot design the data collection process. For instance, missing data, variations in how data are recorded across jurisdictions or sites, and policy changes that altered how data fields were used over time are all common occurrences with administrative data. In addition, the available IDS data may not sufficiently represent the outcome of interest. With multiple sources of administrative data, understanding each dataset and potential quality issues become even more critical for developing sound and rigorous evaluation methodology. Moreover, it is critical for evaluators to approach this work with a strong knowledge of methods for working with large datasets (Santiago & Smith, 2019).

Before getting started, evaluators who plan on linking their own dataset with administrative data should ask IDS administrators what data are helpful to collect. For example, collecting unique identifiers that align with those stored in the IDS will allow for higher quality and easier individual record linkage. Evaluators should also work with the IDS data team to ensure their data are structured and formatted in a way that can be matched with IDS data. This can be achieved by simply asking for an example of the required data structure. Another helpful step is to work with the IDS to perform a match between the IDS data and the evaluator’s data in order to show the number and percentage of overlapping cases. This type of process does not require IRB approval, given that data are not being shared back with the evaluator at this point, but it may require a confidentiality agreement. While it creates some additional work, the insights gained can demonstrate if there is sufficient data to justify the evaluation before going through the longer data sharing process.

### 5.7 Align proposals with the evaluator’s scope of expertise and the priorities and capabilities of the IDS and data contributors

Not all available administrative data will be applicable or appropriate for all evaluation projects. Though the universe of information held within an IDS can be enticing, it is important that evaluators request data that are relevant and specific to their proposal and to their organizational and/or individual expertise. Data requests should be carefully considered and include the minimum data necessary to fulfill the evaluation plan.

A good practice is to make the clear case for why certain types of data are being requested and how those sources contribute to the data model, methods, and other relevant aspects of the proposal. IDS sites want to know how their data will enhance an evaluation. Demonstrating how a proposal fits in with the mission or vision of an IDS can also build trust and generate interest among data sharing agencies who may otherwise lack a financial incentive to work with a lesser-known evaluation firm or to engage in projects outside their purview. Typically, data access requests are reviewed by a committee or group during which data contributing agencies can decide whether or not to share their data for a specific purpose. Agencies want to know that the people represented in their datasets are not at risk if data are shared.^6^ Further, it is important that the evaluator make a clear and concise case up front to help staff determine if a request is financially and technically feasible.

Daunting as it may sound to get approval from each data contributing agency, an IDS offers a direct route to multiple agencies, departments, and sources of information. These practices can facilitate evaluators who do not have a pre-existing relationship with an IDS or their host organization to gain access to restricted data.

### 5.8 Supplement integrated data with survey and other self-report data

This mixed methods approach can shed light on the strengths and weaknesses of both data sources while also providing a more robust evaluation. For instance, integrated data tend to be more accurate when it comes to dates, costs, and types of services received, whereas getting this type of information via self-report is often more time-consuming and less reliable. Administrative records are also less vulnerable to social desirability bias and can be used to omit sensitive survey questions (e.g., Were you involved in the child welfare system? Have you received economic services?). In this way, using administrative data can mitigate the harm to program participants in asking them to relive traumatic experiences during survey and interview data collection, making it a valuable asset to evaluators. Self-report data, however, can capture important experiences, feelings, identities, and protective factors that are not typically documented in administrative records, for example: informal employment wages; social support systems; self-identified race, ethnicity, sexual orientation, and gender identity; and satisfaction with services received. With both integrated data and self-report data, evaluators gain the ability to triangulate findings and test the reliability and validity of each data source.

For evaluators interested in linking survey and other data sources to administrative records (e.g., a specific program list), it is crucial to think through the process well in advance. First, before conducting the survey or other data collection, be clear about the data needed to adequately address the evaluation plan and ensure these data can be linked legally and ethically. For instance, evaluators will need to gain informed consent from participants to link their data with administrative records and use it for evaluation purposes. Second, carefully think through the linkage process. External evaluators will typically only receive deidentified administrative records from an IDS, so any linking would need to be conducted by the IDS prior to creating the analytic data set. The release of identifiable records may be legally allowable if informed consents are received from study participants, but this is beyond the scope of procedures for most IDS sites.

If an informed consent is used, it is important to honor the spirit of informed consent while also being practical. Asking participants to review a long, jargon-filled checklist of data sources may be confusing and counterproductive. Being overly broad and unspecific about data sources can also undermine the ability to get true informed consent. Instead, use terms that are easily understandable to describe the nature of the data sources for potential linkage, and only include one place for the respondent to indicate whether or not they approve rather than placing “yes” or “no” checkboxes next to each type of administrative data source. Additionally, evaluators may need to collect permissions more than once for some participants (e.g., a respondent may have been a minor when the study began, meaning they need to be re-consented upon reaching the legal age of adulthood).

### 5.9 Design and propose evaluations that clearly benefit the communities and/or agencies under evaluation

When using IDS data there are multiple data partners impacted, which heightens the level of planning, diligence, and accountability needed to ensure mutual benefit. All proposals and evaluation questions should establish clear user need for data and public benefit as a result of the project (Scott et al., 2018). Beyond that, evaluators can serve communities and/or agencies by sharing data analyses and reports back with all partners, hosting public events to share preliminary findings and receive community feedback, and ensuring findings highlight tangible changes or information that can directly support the impacted populations. In order to understand how to give back to communities or agencies, ask and listen (Future of Privacy Forum & Actionable Intelligence for Social Policy, 2018). Any provisions should be established up front along with a structure to hold all parties accountable to such agreements.

In that same vein, research is too often conducted in a vacuum without the communities impacted knowing about it or the findings. Therefore, it is important that evaluators involve communities represented in the data when planning projects as a way of assessing implications for racial equity or potential disparate impact on marginalized groups. Additionally, community input can be sought during the analysis, reporting, and dissemination phases as a way to center racial equity, build trust, mitigate potential harm, and improve the usability and relevance of findings (Hawn Nelson et al., 2020a). Further, a lack of centralized records of research and evaluation in local communities diminishes the value and use of historical knowledge. Establishing standards and practices that ensure findings are not only in the hands of researchers, but also agencies and communities, can move findings from knowledge to action, reduce unnecessary evaluations, and build on prior studies. Evaluators can inquire about past research and findings from their local IDS and seek to understand if the system or organization can act as a repository for community-based data and knowledge.

## 6 Work in action: Examples of integrated data used in evaluation

The following examples highlight “Work in Action” – IDS sites and evaluators who have used integrated administrative data to evaluate programs. These examples illustrate the potential payoff of working with IDS, particularly when evaluating complex social issues and seeking longitudinal insights.

### 6.1 Evaluation of Philadelphia’s Kensington Encampment Resolution Pilot, May 2018–March 2019

The City of Philadelphia funded a team from the University of Pennsylvania’s School of Social Policy & Practice to evaluate the City’s Encampment Resolution Pilot (ERP), an initiative to shut down two homeless encampments in the Kensington area. The closures were part of a city-wide effort to combat the growing opioid crisis by connecting people with addiction to housing and substance use treatment services. Analysts from the IDS linked service record data from six agencies contained in the City’s IDS with a comprehensive by-name list of all persons eligible to receive services under the ERP. The evaluators received aggregated results of the record linkage, allowing them to evaluate the extent to which the target population—people sleeping in encampments—received housing and substance use services prior to, during, and 4.5 months after the ERP. Evaluators supplemented these data by interviewing and surveying stakeholders, observing City meetings, and examining relevant city records and media coverage to gain a holistic picture of the ERP’s impact. The final report examines outcomes for those who were displaced by the encampment closures as well as the impact on the broader community and presents key findings in 22 “lessons learned” (Metraux et al., 2019).

### 6.2 Program evaluation: McClintock partners in education, 2007–2015

During the spring of 2014, the Charlotte-Mecklenburg School System (North Carolina) and donors from the McClintock Partners in Education program (McPIE) funded a team from the UNC Charlotte Urban Institute for a yearlong evaluation of McPIE’s impact on McClintock Middle School students’ academic and behavioral outcomes. McPIE is a joint effort between McClintock Middle School, the Christ Lutheran Church, and community volunteers to provide students with the best educational experience through access to support, opportunities, and resources. Researchers from the Urban Institute matched data from Charlotte-Mecklenburg Schools with data from the Department of Social Services Youth and Family Services. The integrated data was supplemented by interviews with students, families, faculty, staff, and volunteers, and observation protocols of the in-person programming. The mixed-methods approach allowed researchers to evaluate the efficacy of this public-private partnership and its impact on the attendance, chronic absenteeism, behavior records, academic performance, and literacy rates of McClintock students. The use of administrative data allowed for two demographically matched comparison points and a longitudinal study of lasting outcomes during high school. The final report evaluates the outcomes associated with participation in each individual aspect of the McPIE program (Gavarkavich et al., 2015).

### 6.3 South Carolina DHHS nurse family partnership expansion and pay for success evaluation from J-PAL North America: 2016–2020

An ongoing evaluation of the South Carolina Department of Health and Human Services’ innovative expansion of the Nurse Family Partnership (NFP)—a long-standing home-visiting program—uses an individual-level RCT design to assess the program’s short- and long-term effectiveness. Over the course of four years, independent evaluators from Jameel-Poverty Action Lab (J-PAL) North America have observed outcomes of 4000 low-income, first-time mothers and their children receiving NFP support in comparison to the control group of 2000. Administrative data from the South Carolina Department of Health and Environmental Control and the South Carolina Data Oversight Council have been used to “evaluate the average impact of NFP on preterm birth, birth spacing, child injury, as well as the long-term health, education, and economic self-sufficiency of the family” (McConnell et al., 2020, n.p.). The project, funded by both federal and state Medicaid dollars as well as philanthropic funders through a Pay For Success contract, is the largest RCT trial of NFP to date.^7^

## 7 How to access administrative data for integration

While some IDS sites are closed to external researchers and evaluators, Table 1 shows sites with publicly available pathways for external parties to potentially work with their data. A few sites denoted below are still developing their processes but expect to have public-facing protocols in the near future.

**Table 1:** IDS sites with public-facing data request processes for external evaluators (as of August 2021).

IDS Site Name	Data Holdings	Management Model	Public Link
Allegheny County Data Warehouse	Child Welfare Early Childhood Economic Security Education Health Housing/Homelessness Justice Vital Records	Agency	https://www.alleghenycountyanalytics.us/index.php/requesting-data/
Baltimore Youth Data Hub	Education Health Public Safety Economic Security	Non-Profit	*Process in development* https://www.baltimorespromise.org
Georgia Cross-Agency Child Data System (CACDS)	Early Childhood	Agency	See CACDS Policy Manual, available at: http://www.gacacds.com/About.aspx
Hartford Data Collaborative (HDC)	Education Non-profit Workforce development Child Welfare	Non-Profit / Public Partnership	https://www.ctdata.org/data-license-request-process
Indiana Management Performance Hub (MPH)	Child Welfare Economic Security Education Health Justice Vital Records	Executive	https://www.in.gov/mph/request-data/
Institute for Social Capital (ISC)	Child Welfare Early Childhood Housing/Homelessness Justice K-12 Education Mental Health Non-Profit	University-Public Partnership	https://ui.uncc.edu/our-work/institute-social-capital/accessing-isc-data
Iowa’s Integrated Data System for Decision-Making (I2D2)	Vital Records Health Early Childhood Education	University-Public Partnership	See Project Requirements and Governance plan, available at: https://i2d2.iastate.edu/governance/
Kentucky Center for Statistics (KYSTATS)	Education Workforce	Agency	https://kystats.ky.gov/Reports/DataRequest
Linked Information Network of Colorado (LINC)	Child Welfare Housing/Homelessness Justice Vital Records	University-Public Partnership	https://coloradolab.org/working-with-the-colorado-lab/call-for-ideas/ Note: proposed ideas must be submitted through State agency staff partner
Miami-Dade IDEAS Consortium for Children	Early Childhood Education	University-Public Partnership	*Process in development* https://ideas.psy.miami.edu
North Carolina Early Childhood Integrated Data System (NC ECIDS)	Child Welfare Early Childhood Education Health Vital Records	Agency	See section on using NC ECIDS data: https://www.ncdhhs.gov/about/department-initiatives/early-childhood/north-carolina-early-childhood-integrated-data-system
North Carolina Education Research Data Center	Education *Information on data holdings found at https://childandfamilypolicy.duke.edu/wp-content/uploads/2019/08/Data-Summaries_July2019.pdf	University-Public Partnership	See Procedures for Obtaining Data: https://childandfamilypolicy.duke.edu/north-carolina-education-research-data/
Ohio Longitudinal Data Archive (OLDA)	Education Workforce Development	University-Public Partnership	https://ohioanalytics.gov/DataAccess/PDF/DataAccess.stm
Philadelphia Office of Integrated Data for Evidence and Action (IDEA)	Child Welfare Early Childhood Health Housing/Homelessness Justice Vital Records	Agency	https://www.phila.gov/departments/office-of-integrated-data-for-evidence-and-action/
South Carolina Integrated Data System	Child Welfare Early Childhood Economic Security Health Housing Vital Records Justice	Executive	http://rfa.sc.gov/healthcare/dataoversight
Texas Education Research Center (ERC)	Education *Information on data holdings found at: https://texaserc.utexas.edu/erc-data/	University-Public Partnership	https://texaserc.utexas.edu/proposal-preparation-submission/

Unless otherwise noted, information about site-specific data holding categories was pulled from AISP's Data Sharing Landscape tool available at: https://www.aisp.upenn.edu/data-sharing-landscape/.

This list is based upon AISP’s most recent national survey and information from the AISP data sharing landscape tool,^8^ publicly available documents, and internal knowledge as of August 2021. This list is not intended to be exhaustive, as the field is continually evolving. The management model is identified for each site, which as noted previously, will shape the evaluator’s approach. It is also important to note when reading this table that access points differ across sites. Sites may require an internal sponsor, monetary fee, and/or extensive approval process in order to access data. This table is merely a starting point, after which the previous section on “How to Work with Integrated Data Systems” may serve as a guide for how to best proceed with approaching and working with sites.

## 8 Conclusion

This article documents the history of data integration efforts in the U.S. and provides recommendations for proposing meaningful evaluation projects and creating long-term partnerships with IDS sites. Evaluations of the Philadelphia Encampment Resolution Pilot, McClintock Middle School student outcomes, and South Carolina’s Nurse Family Partnership program highlight the ways in which IDS data can strengthen evaluation efforts. Finally, a list of IDS sites with public-facing processes for external data requests is provided in order to foster awareness and potential collaborations within the field.

The field of data integration has evolved since the early work of linking vital statistics records— there are now over 90 documented data integration efforts in the U.S. alone. Despite the increasing use of linked data for evaluation, hurdles remain, including unclear data access processes and challenges communicating mutual benefit for evaluators and data contributing agencies. In addition, evaluators must address data quality concerns within administrative datasets and think critically about how to operationalize variables represented by data not collected for evaluation purposes. Despite these challenges, the benefits of linking administrative data for evaluation are numerous, as doing so unearths key understandings about how policies and programs impact the whole person or family and provides decision-makers with actionable intelligence on what works and for whom. This article intends not only to point evaluators to opportunities to elevate their work with integrated data, but also to encourage IDS sites to create sustainable pathways to leverage the skill and expertize of external evaluators. With an IDS in place, data integration staff and external evaluators can partner to enhance the field of social policy evaluation through strategic collaboration and evidence-building.

## Funding

This work was supported by the 10.13039/100000865Bill & Melinda Gates Foundation, Seattle, WA [Grant number INV-003672].

## CRediT authorship contribution statement

**Sharon Zanti:** Conceptualization, Methodology, Investigation, Project administration, Writing – original draft, Writing – review & editing. **Emily Berkowitz:** Data curation, Investigation, Methodology, Validation, Writing – original draft, Writing – review & editing. **Matthew Katz:** Writing – review & editing, Investigation, Visualization. **Amy Hawn Nelson:** Investigation, Data curation, Writing – review & editing. **T.C. Burnett:** Conceptualization, Supervision, Writing – review & editing. **Dennis Culhane:** Funding acquisition, Supervision, Writing – review & editing. **Yixi Zhou:** Writing – review & editing.

## Conflict of interest statement

The authors declare they have no conflicts of interest.

## Data Availability

The unpublished report produced for the Gates Foundation, “IDS Landscape Analysis: Models, motivations, and capacity for cross-agency data sharing” (2020), is available upon request and provides aggregate data from the national survey of integrated data efforts. Data holdings for IDS sites listed in Table 1 can be found primarily on the AISP Data Sharing Landscape Tool: https://www.aisp.upenn.edu/data-sharing-landscape/. In addition, AISP maintains a Resource Library (located here: https://www.aisp.upenn.edu/resource-library/), which contains much of the internal expertise drawn upon for this article.

## Footnotes

1 Data integration is “a complex type of data sharing that involves record linkage, which refers to the joining or merging of data based on common data fields.” (Hawn Nelson et al., 2020b, p.1).

2 Governance and legal structures for IDS are ideally developed collaboratively and foster trust, cooperation, and engagement across all data sharing agencies and partners (Actionable Intelligence for Social Policy, 2021).

3 The eight sites identified through the national landscape scan were: the State of Michigan, State of South Carolina, County of Los Angeles, Allegheny County in Pennsylvania, University of South Florida (for the State of Florida), University of Pennsylvania (for the City of Philadelphia), University of Chicago (for the State of Illinois), and Case Western Reserve University (for Cuyahoga County, OH) (Culhane et al., 2010).

4 Administrative datasets represent people who are disproportionately living in poverty, and, as a result of the historical legacy of race in America, disproportionately represent Black, Indigenous, and people of color (BIPOC). As such, "seeing these data as race-neutral is inaccurate, and such views could lead to system-level data use that unintentionally replicates structural racism” (Hawn Nelson et al., 2020a, p.3).

5 Social license refers to the broad social acceptance and approval IDS sites must develop in order to have lasting success. Social license is established through the IDS’s legitimacy, credibility, compliance with legal and privacy rules, and overall public trust. For more information on developing social license, see https://fpf.org/wp-content/uploads/2018/09/FPF-AISP_Nothing-to-Hide.pdf (Future of Privacy Forum & Actionable Intelligence for Social Policy, 2018).

6 See *A Toolkit for Centering Racial Equity Throughout Data Integration* for a more nuanced discussion of balancing risk versus benefit: https://www.aisp.upenn.edu/centering-equity/.

7 A full list of partners, including project consultants, is available here: https://www.scdhhs.gov/sites/default/files/2-16-16-SC-NFP-PFS-Partners_0.pdf.

8 See https://www.aisp.upenn.edu/data-sharing-landscape/.
